# Pre-pregnancy check-up of maternal vascular status and associated phenotype is crucial for the health of mother and offspring

**DOI:** 10.1007/s13167-022-00294-1

**Published:** 2022-08-17

**Authors:** Maria Evsevieva, Oksana Sergeeva, Alena Mazurakova, Lenka Koklesova, Irina Prokhorenko-Kolomoytseva, Evgenij Shchetinin, Colin Birkenbihl, Vincenzo Costigliola, Peter Kubatka, Olga Golubnitschaja

**Affiliations:** 1 Stavropol State Medical University, Stavropol, Russian Federation; 2 Clinic of Obstetrics and Gynecology, Jessenius Faculty of Medicine, Comenius University in Bratislava, 036 01 Martin, Slovakia; 3 Fraunhofer Institute Bonn, Bonn, Germany; 4 European Medical Association, EMA, Brussels, Belgium; 5 Department of Medical Biology, Jessenius Faculty of Medicine, Comenius University in Bratislava, 036 01 Martin, Slovakia; 6 Predictive, Preventive and Personalised (3P) Medicine, Department of Radiation Oncology, University Hospital Bonn, Rheinische Friedrich-Wilhelms-Universität Bonn, 53127 Bonn, Germany

**Keywords:** Predictive preventive personalised medicine (PPPM / 3PM), Pregnancy, Health-to-disease transition, Body mass index, Bodyweight, Sub-optimal health, Flammer syndrome phenotype, Systemic effects, Mother, Offspring, Risk assessment, Cardiovascular stress, Maladaptation, Ischemic stroke, Wound healing, Vascular stiffness, Blood pressure, Connective tissue dysfunction, Mitral valve prolapse, Anthropometry, Hypotrophy, Preterm birth, Antenatal foetal death, Preeclampsia, Gestational arterial hypertension, Gestational diabetes, Oligohydramnios, Foetal growth restriction, Phytochemicals, Individualised protection, Primary care, Pre-pregnancy check-up, Young populations, Population screening, Health policy

## Abstract

**Abstract:**

Cardiovascular disease remains the leading cause of disease burden globally with far-reaching consequences including enormous socio-economic burden to healthcare and society at large. Cardiovascular health is decisive for reproductive function, healthy pregnancy and postpartum. During pregnancy, maternal cardiovascular system is exposed to highly increased haemodynamic stress that significantly impacts health status of the mother and offspring. Resulting from sub-optimal maternal health conditions overlooked in pre-pregnancy time, progressive abnormalities can be expected during pregnancy and postpartum. Contextually, there are two main concepts to follow in the framework of predictive, preventive and personalised medicine, namely to develop:

1. advanced screening of sub-optimal health conditions in young populations to predict and prevent individual health risks prior to planned pregnancies

2. in-depth companion diagnostics during pregnancy to predict and prevent long-lasting postpartum health risks of the mother and offspring.

Data collected in the current study demonstrate group-specific complications to health of the mother and offspring and clinical relevance of the related phenotyping in pre-pregnant mothers. Diagnostic approach proposed in this study revealed its great clinical utility demonstrating important synergies between cardiovascular maladaptation and connective tissue dysfunction. Co-diagnosed pre-pregnancy low BMI of the mother, connective tissue dysfunction, increased stiffness of peripheral vessels and decreased blood pressure are considered a highly specific maternal phenotype useful for innovative screening programmes in young populations to predict and prevent severe risks to health of the mother and offspring. This crucial discovery brings together systemic effects characteristic, for example, for individuals with Flammer syndrome predisposed to the phenotype-specific primary vascular dysregulation, pregnancy-associated risks, normal tension glaucoma, ischemic stroke at young age, impaired wound healing and associated disorders. Proposed maternal phenotyping is crucial to predict and effectively protect both the mother and offspring against health-to-disease transition. Pre-pregnancy check-up focused on sub-optimal health and utilising here described phenotypes is pivotal for advanced health policy.

**Plain English abstract:**

Cardiovascular health is decisive for reproductive function and healthy pregnancy. During pregnancy, maternal cardiovascular system may demonstrate health-to-disease transition relevant for the affected mother and offspring. Overlooked in pre-pregnancy time, progressive abnormalities can be expected during pregnancy and lifelong. Here we co-diagnosed maternal pre-pregnancy low bodyweight with systemic effects which may increase risks of pregnancy, eye and heart disorders and ischemic stroke at young age, amongst others. Innovative screening  programmes focused on sub-optimal health in young populations to predict and to mitigate individual health risks prior to pregnancy is an essential innovation for health policy proposed.

## Introduction

### Cardiovascular health issues in young populations with far-reaching consequences

Cardiovascular disease (CVD) remains the leading cause of disease burden globally with far-reaching consequences including enormous socio-economic burden to healthcare and society at large. In particular, ischemic heart disease and stroke represent the leading cause of mortality worldwide being the major contributor to disability. According to the global statistics presented [1], CVD prevalence nearly doubled from 271 million in 1990 to 523 million in 2019. Corresponding numbers of the CVD-related deaths were steadily increasing from 12,1 million (1990) to 18,6 million (2019). Over that period of time also disability doubled from 17,7 million to 34,4 million. To this end, alarming statistics demonstrate rapidly increasing incidence of ischemic stroke of unclear aetiology characteristic specifically for young populations [2]. International multi-professional expert groups strongly recommend implementing cost-effective policies [1] and paradigm change from reactive to predictive, preventive and personalised medicine (PPPM / 3PM) to reduce premature mortality and disability due to preventable non-communicable diseases such as stroke at young age with modifiable risk factors [2–4].

Cardiovascular health is decisive for reproductive function and healthy pregnancy. Gestation is broadly recognised as being critical for both — maternal health and the offspring’s life-long health after birth [5]. To this end, the USA study demonstrated for years 2011–2013 pregnancy-related mortality as high as 17,0 deaths per 100.000 live births in the country; cardiovascular conditions have been ranked first (15,5%) amongst the main risks [6]. Maternal cardiovascular health during pregnancy (five metrics were used, namely BMI, blood pressure, total cholesterol and glucose levels, smoking exposure) has been demonstrated as being strongly associated with offspring cardiovascular health quality during early adolescence (four metrics were used: BMI, blood pressure, total cholesterol and glucose levels). For this study, a multinational cohort included 2.302 mother–child dyads demonstrating the interrelation between poorer maternal cardiovascular health at a mean of 28 weeks’ gestation and high risks of poorer offspring cardiovascular health at ages 10 to 14 years [7].

### Overlooked sub-optimal health may lead to progressive abnormalities in pregnancy and postpartum

During pregnancy, maternal cardiovascular system is exposed to highly increased haemodynamic stress that significantly impacts health status of mother and offspring [8, 9]. Resulting from sub-optimal maternal health conditions overlooked in pre-pregnancy time, progressive abnormalities can be expected in the foetal development and maternal health status during pregnancy and postpartum. To this end, for example, Flammer syndrome (FS) phenotype was described as being of great clinical utility for sub-optimal health risk assessment [10]. Based on the individualised patient profile, FS phenotype is well detectable being linked to low BMI, disturbed microcirculation, abnormal stress reactions, shifted sense regulation and specific behavioural patterns, amongst others [11]. FS phenotyping is fully feasible early in life during pubertal development, in order to assess individual health risks and, later on in life, also to predict potential pregnancy complications followed by targeted mitigating measures [2, 12].

Contextually, there are two main concepts to follow in the context of predictive, preventive and personalised medicine, namely to develop:

1. advanced screening of sub-optimal health in young populations to predict and prevent individual health risks to optimise the course of planned pregnancies [3, 13],
2. in-depth companion diagnostics during pregnancy to predict and prevent potentially lasting postpartum health risks of mother and offspring [4].

To this end, abnormal (both underweight and overweight) BMI which is highly individual anthropometric parameter has been associated with sub-optimal health status and large spectrum of pathologies developed later on in life [3]. Consequently, abnormal vascular parameters such as increased vascular stiffness (VS) were, further, associated with the phenotype-characteristic clinically manifested symptoms of connective tissue dysfunction (CTD) frequently linked to low BMI [14, 15]. Noteworthy, incidence of CTD is increasingly reported specifically for young populations being further attributed to the alterations in human genetic apparatus adapted to changing environmental conditions. This actuality prompts developing innovative screening programmes adapted specifically to the needs of young generations considering advanced predictive diagnostic tools e.g. for planned pregnancies followed by targeted prevention and personalised treatment algorithms tailored to the person at high risk for complications in pregnancy and postpartum.

##  Working hypothesis and study aims in the framework of 3P medicine

In the current study, we hypothesised that vascular status and bodyweight-associated phenotyping might be instrumental to predict potential pregnancy and postpartum risks for personalised supervision of planned pregnancies by prevention tailored to predicted risks. The aim was to stratify pregnant women taking into account maternal pre-pregnancy BMI, external manifestations of CTD exemplified by Flammer syndrome manifestation as clearly detectable phenotype with specific vascular status particularities, low BMI and predisposition to evident risks in pregnancy and postpartum described earlier [12]. Contextually, the study aimed at investigating potential relationship between individual phenotypes, vascular status of pregnant women, frequency of pregnancy complications and corresponding anthropometric parameters of newborns, amongst others.

## Study design

### Patient recruitment and stratification criteria

A total of 345 pregnant women (further called *study participants* abbreviated as SPs) with singleton pregnancies (mean age 27,2 ± 2,5 l) were investigated. All SPs were stratified into three groups of comparison based on the initial (pre-pregnancy) body mass index (BMI) according to the classification published in the EPMA Position Paper 2021 [3]:

group 1 – low BMI (18,9 or less kg/m^2^) with 27 SPs;

group 2 – standard BMI range 19–25 kg/m^2^ with 216 SPs;

group 3 – high BMI (25,1 or more kg/m^2^) with 102 SPs.

### Clinical examinations

Stratified groups of comparison underwent comprehensive examinations including anthropometry (height, weight, BMI), blood pressure measurements, surveys performance covering questions towards history of traditional cardiovascular risk factors (arterial hypertension, hereditary history of early cardiovascular disease, smoking etc.) and history of previous pregnancies (if any), including preeclampsia & gestational arterial hypertension (PE & GAH).

#### Haemodynamic and vascular status parameters

Non-invasive assessment of vascular stiffness for both peripheral small vessels and aorta was performed by oscillometric testing using a BPLab Vasotens (Petr Telegin, Russia) in Vasotens Office format at gestational age up to 20 weeks [16]. The device meets international accuracy standards for oscillometric BP recorders and is recommended for use in pregnant women [17, 18].

Vascular stiffness parameters were measured through peripheral systolic blood pressure (PSBP), peripheral diastolic blood pressure (PDBP), peripheral arterial vascular stiffness (AIXb), aortic reverse pulse wave velocity (RWVa), aortic vascular stiffness (AIXa), central systolic blood pressure (CSBP) and central diastolic blood pressure (CDBP).

#### Connective tissue dysfunction

Functionally linked to the arterial stiffness in young adults connective tissue dysfunction (CTD) was assessed according to the Ghent Diagnostic Criteria Edition 2 [19] and national guidelines [15].

The extent of connective tissue dysfunction was scored. According to the expert recommendations in the area, particular attention was paid to the occurrence of the most specific and therefore diagnostically significant CTD features such as thumb and wrist symptoms, chest deformities, valgus deformity of the foot, flat foot, scoliosis, kyphosis, incomplete (170°) extension of the elbow, myopia, atypical strictures, specific facial features (such as dolichocephaly, enophthalmos, downward sloping eye chambers, malar bone hypoplasia, retrognathia), up/down ratio ≤ 0,86 and arm-span/height ≥ 1,05 without significant scoliosis, mitral valve prolapse and indication of spontaneous pneumothorax, amongst others.

### Phenotyping by Flammer syndrome symptoms and signs

A randomized trial considered 60 SPs for the Flammer’s syndrome (FS) phenotype screening. The specialised questionnaire [10, 12] was utilised for the FS phenotype specific symptoms and signs including low BMI, frequently cold extremities, feeling of chills, low blood pressure, perfectionism, lack of feeling of thirst and hyperemic spots when agitated, amongst others as described earlier [10, 12]. Consequently, the group under investigation was stratified into two cohorts, namely with (FS +) and without (FS −) FS phenotype.

### Pregnancy complications considered

Preeclampsia, gestational arterial hypertension and diabetes were recorded for mothers, while foetal growth restriction, antenatal foetal death and preterm birth were recorded for their offspring. Anthropometric parameters of newborns were used to calculate corresponding BMI.

### Exclusion criteria

The exclusion criteria are administration of drugs affecting blood pressure; specific hereditary syndromes such as Marfan and Ehlers-Danlos, multiple pregnancies and pregnancies which resulted from assisted reproductive technology.

### Statistical analysis

The data were processed using the statistical software package IBM SPSS Statistics 21. The number observations of different diseases were compared across the study groups using pairwise Fisher’s-exact-tests. All tests were performed two-sided and findings were considered statistically significant according to the *P* value below 0,05. We performed a Benjamini–Hochberg correction to account for multiple testing.

## Results

### Interrelationship between pre-pregnancy BMI and incidence of connective tissue dysfunction

**Fig. 1:**
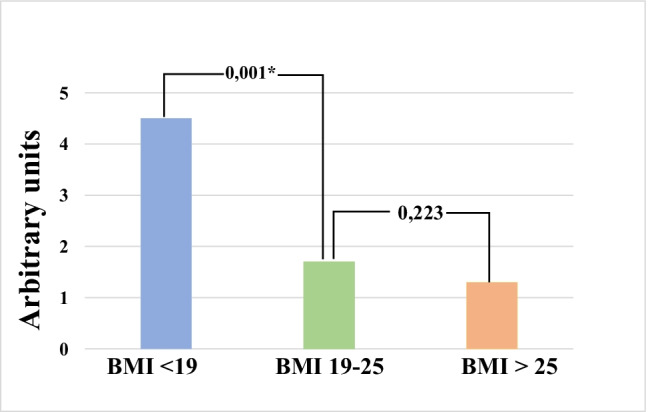
Scoring of the symptoms and signs specific for the connective tissue dysfunction in pregnant women stratified by corresponding pre-pregnancy body mass index (BMI); significance is demonstrated (*P* value)

Data from the phenotypic analysis demonstrated clearly inverse relationship between increasing BMI of SPs (from group 1 to group 3) and decreasing incidence of CTD (see Fig. 1). Whereas a non-significant CTD incidence decrease was demonstrated for high BMI group 3 compared to the group 2 with standard BMI-range, highly significant CTD incidence increase was observed for the group 1 with low BMI. To this end, 30% of SPs in the group 1 demonstrated five and more stigmatic phenotypic features, indicating the presence of non-specific CTD variants [15].

### Interrelation between Flammer syndrome phenotype and incidence of connective tissue dysfunction

**Fig. 2:**
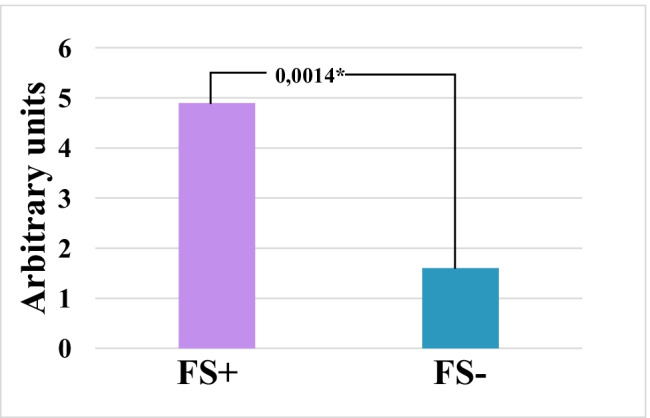
Scoring of the symptoms and signs specific for the connective tissue dysfunction in pregnant women with (FS +) and without (FS −) Flammer syndrome phenotype; significance is demonstrated (*P* value)

An incidence of external signs of hereditary connective tissue disorders in pregnant women with the Flammer syndrome phenotype (FS +) was by a factor of 3,5 significantly higher (*P* = 0,0014) compared with SPs without Flammer syndrome phenotype (FS −) (see Fig. 2).

This discovery may potentially indicate a new highly specific symptom of Flammer syndrome which has not been yet described in the literature and can be potentially linked with systemic effects characteristic for this phenotype (see the “Data interpretation” section provided below).

### Maternal haemodynamic and vascular health status

Data analysis of haemodynamic parameters (Table 1) revealed that both peripheral (PSBP) and central (CSBP) systolic blood pressure increased steadily from 1 to 3 groups of comparison: at the brachial artery by 12,0 mmHg (*P* = 0,0001*) and in the aorta by 9,6 mmHg (*P* = 0,0001*) (see Table 1). Although being statistically non-significant, similar trend was recorded also for diastolic blood pressure (PDBP) increased in the 3rd group compared to both 1 and 2 ones.

**Table 1:** Individual parameters measured to characterise haemodynamic and vascular status of pregnant women stratified by corresponding pre-pregnancy BMI; statistically significant *P* values are presented in bold letters and marked with an asterisk; statistically differences were demonstrated between the group 1 (low BMI) and group 3 (high BMI) specifically for parameters PSBP (113,9 ± 1,6 against 125,9 ± 1,27 mm Hg) and CSBP (101,7 ± 1,4 against 111,1 ± 1,16 mm Hg), as well as between group 2 (normal BMI) and group 3 (high BMI) specifically for parameters PSBP (116,1 ± 0,7 against 125,9 ± 1,27 mm Hg), RWVa (9,4 ± 0,1 against 10,1 ± 0,19 m/s), CSBP (103,6 ± 0,68 against 111,1 ± 1,16 mm Hg) and AIXa (3,08 ± 0,46 against 4,28 ± 0,41)

Individual parameters	BMI < 19 kg/m^2^ *n* = 27 Group 1	BMI ranged 19–25 kg/m^2^ *n* = 216 Group 2	BMI > 25 kg/m^2^ *n* = 102 Group 3	Significance *P* Groups 1, 2, 3 of comparison
Peripheral systolic blood pressure (PSBP), mm Hg	113,9 ± 1,6	116,1 ± 0,7	125,9 ± 1,27	*P1-2* = *0,23* ***P1-3*** = 0,0001* *P2-3* = 0,0001*
Peripheral diastolic blood pressure (PDBP), mm Hg	72,3 ± 1,5	72,8 ± 0,5	75,8 ± 1,1	*P1-2* = *0,75* *P1-3* = *0,069* *P2-3* = *0,16*
Peripheral arterial vascular stiffness (brachial augmentation index, AIXb), %	− 32,5 ± 1,51	− 49,7 ± 1,0	− 44,4 ± 1,53	*P1-2* = 0,032* *P1-3* = *0,071* *P2-3* = *0,344*
Aortic reverse pulse wave velocity (RWVa), m/s	9,7 ± 0,26	9,4 ± 0,1	10,1 ± 0,19	*P1-2* = *0,193* *P1-3* = *0,062* *P2-3* = 0,002*
Central systolic blood pressure (CSBP), mm Hg	101,7 ± 1,4	103,6 ± 0,68	111,1 ± 1,16	*P1-2* = *0,248* *P1-3* = 0,0001* *P2-3* = 0,0001*
Central diastolic blood pressure (CDBP), mm Hg	74,3 ± 1,5	74,9 ± 0,5	77,9 ± 1,05	*P1-2* = *0,691* *P1-3* = *0,06* *P2-3* = *0,15*
Aortic vascular stiffness (aortic augmentation index, AIXa) %	3,32 ± 0,54	3,08 ± 0,46	4,28 ± 0,41	*P1-2* = *0,643* *P1-3* = *0,177* *P2-3* = 0,026*

Peripheral arterial vascular stiffness AIXb demonstrated the highest values in group 1 (low BMI), while AIXa (aortic vascular stiffness) and RWVa (aortic reverse pulse wave velocity) were significantly higher in group 3 (overweight). Both AIXa and RWVa values were non-significantly higher in group 1 compared to group 2.

### Anthropometric status of newborns in 3 groups of comparison

Anthropometric status of newborns reflecting their nutritional status in the antenatal period demonstrated that the number of low-birth-weight babies declined steadily from group 1 to group 3. In contrast, the number with normal and excessive body weight increased from group 1 to group 3. BMI below 12 kg/m^2^ was characteristic (about 89%) for newborns in group 1, whereas BMI ≥ 12 kg/m^2^ was characteristic (84%) for group 3 (see Table 2).

**Table 2:** Anthropometric parameters of newborns in groups of comparison stratified by corresponding BMI of their pre-pregnant mothers; statistically significant *P* values are presented in bold letters and marked with an asterisk; statistically significant differences were observed, in particular, for absolute majority of hypotrophic newborns (about 78%) in the group 1 (low BMI of pre-pregnant mothers) associated with low BMI < 12 kg/m^2^ in about 89% of all newborns in this group of comparison

Anthropometric (height to weight) parameters of newborns	BMI < 19 kg/m^2^ *n* = 27 % (absolute number) Group 1	BMI ranged 19–25 kg/m^2^ *n* = 216 % (absolute number) Group 2	BMI > 25 kg/m^2^ *n* = 102 % (absolute number) Group 3	Significance *P* Groups 1, 2, 3 of comparison
Hypotrophy	77,8 (21)	25,2 (52)	13,7 (14)	*P1-2* < 0,0001* *P1-3* < 0,0001* *P2-3* = *0,052*
Normotrophy	11,1 (3)	54,2 (118)	68,0 (62)	*P1-2* < 0,0001* *P1-3* < 0,0001* *P2-3* = *0,357*
Hypertrophy	11,1 (3)	25,0 (46)	25,5 (26)	*P1-2* = *0,357* *P1-3* = *0,160* *P2-3* = *0,473*
BMI < 12 kg/m^2^	88,9 (24)	33,3 (72)	15,7 (16)	*P1-2* < 0,0001* *P1-3* < 0,0001* *P2-3* = 0,0018*
BMI ≥ 12 kg/m^2^	11,1 (3)	66,7 (144)	84,3 (86)	*P1-2* < 0,0001* *P1-3* < 0,0001* *P2-3* = 0,0018*

### Maternal and foetal complications recorded in groups of comparison

Overweight women suffered significantly more often from preeclampsia & gestational arterial hypertension (*P* = 0,005*). No cases of preeclampsia and gestational arterial hypertension were registered in group 1 with low BMI. Similarly no cases of gestational diabetes were registered in group 1, whereas overweigh pregnant women in group 3 were suffering from this disorder twice as frequently as their counterparts in group 2 (standard BMI range). Preterm birth was registered solely in group 3. Insufficient maternal weight gain during pregnancy was more characteristic for group 1 (low BMI), whereas excessive maternal weight gain was characteristic for the majority of women (about 55%) in group 3 (overweight).

Collected data demonstrated foetal growth restriction as the most characteristic for group 1 (see Table 3). This complication was less common for group 2 (standard BMI range) and did not occur at all in group 3 (overweight, *P1-3* = 0,038*). Antenatal foetal death was registered solely in group 1.

**Table 3:** Pregnancy complications and weight gain particularities observed in 3 groups of comparison stratified by corresponding BMI of pre-pregnant mothers; antenatal foetal death was observed solely in group 1 (low BMI), whereas preterm birth was observed only in group 3 (high BMI); preeclampsia, gestational arterial hypertension and gestational diabetes were more frequent in group 3, in contrast to foetal growth restriction monitored in group 1 only; insufficient maternal weigh gain was more characteristic for group 1 compared with other group; excessive maternal weight gain was characteristic for group 3; statistically significant *P* values are presented in bold letters and marked with an asterisk

Pre- and peri-natal complications registered	BMI < 19 kg/m^2^ *n* = 27 % (absolute numbers) group 1	BMI range 19–25 kg/m^2^ *n* = 216 % (absolute numbers) group 2	BMI > 25 kg/m^2^ *n* = 102 % (absolute numbers) group 3	Significance *P* Groups 1, 2, 3 of comparison
Antenatal foetal death	3,7 (1)	–	–	*P1-2* = *0,187* *P1-3* = *0,311*
Preterm birth	–	–	3,9 (4)	*P1-3* = *0,693* *P2-3* = *0,039*
Preeclampsia and gestational arterial hypertension	–	4,6 (10)	16,7 (17)	*P1-2* = *0,693* *P1-3* = *0,066* *P2-3* = 0,005*
Gestational diabetes	–	5,1 (11)	10,8 (11)	*P1-2* = *0,693* *P1-3* = *0,188* *P2-3* = *0,175*
Foetal growth restriction FGR	11,1 (3)	1,9 (4)	–	*P1-2* = *0,067* *P1-3* = 0,038* *P2-3* = *0,418*
No complications observed	85,2 (23)	88,4 (191)	68,6 (70)	*P1-2* = *0,694* *P1-3* = *0,176* *P2-3* = 0,0003*
Insufficient weight gain	33,3 (9)	29,2 (63)	13,7 (14)	*P1-2* = *0,712* *P1-3* = *0,669* *P2-3* = *0,164*
Normal weight gain	59,3 (16)	44,9 (97)	31,4 (32)	*P1-2* = *0,311* *P1-3* = *0,043* *P2-3* = *0,067*
Excessive weight gain	7,4 (2)	25,9 (56)	54,9 (56)	*P1-2* = *0,067* *P1-3* < 0,0001* *P2-3* < 0,0001*

### In-depth analysis of characteristic individual cases

#### Case 1

A 23-year-old woman; professional occupation: a nurse at a city medical unit. First pregnancy, no unhealthy behavioural habits recorded. BMI = 15,8 kg/m^2^. Foci of infection: chronic maxillary sinusitis; No family history of CVD.

Her mother’s pregnancy was without complications, and she was born at term weighing 2.600 g. Female medical history: menarche at the age of 12 years.

Flammer syndrome phenotype demonstrates following strongly pronounced symptoms and signs:

- The patient was always very slim with difficulty to gain the weight; she obviously tends to perfectionism
- Frequently cold extremities, she is freezing even during warm seasons
- Pre-pregnancy hypotension, further strongly pronounced early in pregnancy; symptoms of orthostatic hypotension, further strongly pronounced during pregnancy
- Frequent dizziness and pre-syncopal conditions (lipotemia)
- Difficulties to fall asleep; night sleep duration 6–8 h with shifted sleep patterns towards morning hours accompanied with chronic fatigue upon awakening in the morning
- She does not feel thirsty, but by mind controls liquid intake against nausea
- Frequent headache, sometimes with aura.

Manifestations of CTD: extended external stigmatisation including thumb and wrist symptoms, amongst others; mitral valve prolapse of moderate haemodynamic significance; a single supraventricular extra-systole according to a single ECG.

Angiological screening resulted in

- peripheral systolic to diastolic blood pressure 106/66 mm Hg and central systolic to diastolic blood pressure 91/68 mm Hg,
- RWVa 9,3 m/s, AIXb − 31,1%, AIXa 3,1%.

Pregnancy course: The pregnancy was complicated by the development of placental insufficiency; the foetal growth restriction of middle severity (grade 2).

Urgent delivery (37 weeks), newborn weight of 2.480 g, height of 48 cm with corresponding BMI = 10,4 kg/m^2^ (hypotrophy).

Maternal weight gain during pregnancy was 11 kg considered as insufficient.

#### Case 2

A 25-year-old woman; professional occupation: a designer at the private enterprise; academician with University education diploma.

First pregnancy, no unhealthy behavioural habits; BMI = 19,9 kg/m^2^; foci of infection: none.

No family history of CVD. Her mother’s pregnancy was without complications, and she was born at term weighing 3.100 g. Female medical history: menarche at the age of 13 years.

Flammer syndrome phenotype demonstrates following strongly pronounced symptoms and signs:

- Since adolescence there was a tendency to low blood pressure and frequent dizziness, in particular when suddenly standing up
- Perfectionist frequently suffering from severe psycho-emotional stress
- When agitated she often registers red and white spots on her face and neck
- Slowed and impaired wound healing
- Although she preferred warm clothes before pregnancy, the feeling of freezing got extreme during the pregnancy. Especially in the legs, she also noted a feeling of “creeping” and tingling of the fingertips
- Frequent headache during the pregnancy.

Manifestations of CTD: significantly elevated external stigmatisation, especially on the craniofacial segment; thumb and wrist symptoms and scoliosis. Abdominal ultrasound revealed an overstretch of the gallbladder.

Angiological screening resulted in

- peripheral systolic to diastolic blood pressure 108/70 mm Hg and central systolic to diastolic blood pressure 99/72 mm Hg
- RWVa 9,1 m/s, AIXb − 25,9%, AIXa 3,0%.

Pregnancy course: evident oligohydramnious diagnosed with ultrasound. The pregnancy was complicated by the development of placental insufficiency; chronic fetoplacental insufficiency; disordered uterine-foetal-placental blood flow; the foetal growth restriction grade 1 (minor).

Urgent delivery (38 weeks), the newborn weight of 2.980 g and 51 cm of height with corresponding BMI = 11,46 kg/m^2^ (hypotrophy).

Maternal weight gain during pregnancy was 10 kg considered as insufficient.

#### Case 3

A 26-year-old woman; professional occupation: a college teacher; academician with University education diploma.

BMI = 31,6 kg/m^2^; foci of infection: none.

Family history: aggravated by arterial hypertension (mother). She believes that she was overfed since childhood due to low birth weight.

Her mother’s pregnancy was without complications, and she was born at term weighing 2.700 g.

Female medical history: menarche at age 15.

Manifestations of CTD: None

No manifestations of the Flammer syndrome phenotype.

Angiological screening resulted in

- peripheral systolic to diastolic blood pressure 122/78 mm Hg and central systolic to diastolic blood pressure 114/80 mm Hg
- RWVa 10,4 m/s, AIXb − 42,2%, AIXa 4,6%.

The course of pregnancy: no abnormalities.

Urgent delivery (38,5 weeks), newborn weight of 3.780 g, height of 43 cm with corresponding BMI 13,2 kg/m^2^ (hypertrophy).

Maternal weight gain during pregnancy was 13 kg considered as excessive.

## Data interpretation

### Presented results fully support working hypothesis of the study

Presented results fully confirm the working hypothesis indicating that depending on the pre-pregnancy BMI of study participants in groups of comparison, there are clear phenotype-specific differences.

1. in their haemodynamic and vascular status,
2. incidence and severity level of CTD,
3. spectrum of maternal and foetal complications and
4. anthropometric parameters of newborns as detailed below.

### Maternal haemodynamics

Haemodynamic status of pregnant women with both low and high BMI differed significantly from this of women in the group with standard BMI range. Moreover, the difference was characterised as being highly specific for each group of comparison. In particular, for group 1 (low BMI), the most significant increase in vascular stiffness was monitored specifically for small peripheral vessels, while aortic stiffness was affected to much lesser extent. Noteworthy, both systolic (PSBP, CSBP values) and diastolic (PDBP, CDBP values) blood pressure in group 1 were decreased compared to group 2 (standard BMI range). In contrast, group 3 (overweight) demonstrated significantly increased aortic stiffness (RWVa and AIXa values) as well as central systolic blood pressure compared to group 2.

### Maternal connective tissue dysfunction

CTD was demonstrated as being characteristic for group 1 (low BMI) with their most specific manifestations (thumb and wrist symptoms, chest deformities, valgus deformity of the foot, flat foot, scoliosis, kyphosis, myopia and specific facial signs, mitral valve prolapse and indication of spontaneous pneumothorax, amongst others) scored significantly higher for this group compared to group 2 (standard BMI range). These findings are consistent with observations published earlier demonstrating an increased incidence of CTD with signs of nonspecific variants characteristic for individuals with low BMI [10, 12, 20].

Connective tissue dysfunction may be associated with the specific morphology of the vessel walls linked to an increased stiffness of small vessels — both characteristic for low BMI individuals clearly demonstrated for extreme cases such as the Marfan, Loeys-Dietz and Ehlers-Danlos syndromes, and nonspecific connective tissue disorders [14, 21]. This functional link becomes particularly evident under the extreme vascular stress condition such as pregnancy.

### Flammer syndrome individuals — the group of risk with well described phenotype

Noteworthy, data collected in the current study demonstrated CTD as being particularly characteristic for participants with the Flammer syndrome phenotype. Therefore, current study brings together systemic effects characteristic for individuals with FS phenotype — all potentially linked to connective tissue dysfunction and primary vascular dysregulation with increased risks of myopia, normal tension glaucoma, small vessel disease, ischemic stroke at young age, mitral valve prolapse, impaired wound healing and pathological scarring, metastatic cancer and complications in pregnancy, amongst others [2, 3, 10, 12, 22–26]. Contextually, an increased endothelin-1 concentration in blood plasma is characteristic for the Flammer syndrome phenotype that can cause vascular stiffness, particularly in peripheral small vessels and in a long-term way may lead to endothelial dysfunction, low-grade inflammation and related pathologies [4].

### Risks characteristic for maternal pre-pregnancy overweight

Of completely different nature is the mechanism of vascular stiffness and endothelial dysfunction in obese individuals reflected in big vessels (aortic) stiffness linked to increased systolic and diastolic blood pressure as clearly demonstrated in our study under the vascular stress condition during pregnancy. This group was generally free of CTD symptoms and signs. These results are well in consensus with data published by other research groups [27, 28].

It has been demonstrated adipokines play a major role in increasing arterial stiffness in obesity: adipokine dysregulation may cause endothelial dysfunction, vascular wall inflammation and remodelling, which altogether synergistically contribute to increased arterial stiffness [29–31].

### Type and incidence of pregnancy complications are specific for individual phenotypes: far-reaching consequences

Collected data demonstrated group-specific complications in mother and offspring and high relevance of BMI in pre-pregnant mothers recorded. In the literature dedicated to the topic, a particular interest is currently dedicated to preeclampsia and gestational arterial hypertension (PE & GAH) [32–34].

Consequently, in patients with preeclampsia an increased vascular stiffness was demonstrated from the beginning of the second trimester of pregnancy followed by an increase in the aortic augmentation index and reverse pulse wave velocity reflecting a pronounced arterial stiffness. Accompanied with possible left ventricular dysfunction, this indicates that placental dysfunction may occur secondary to maternal cardiovascular maladaptation characteristic for pregnant women diagnosed with preeclampsia [35, 36].

To this end, prospective studies [37, 38] investigating women diagnosed with preeclampsia 6 and 12 months postpartum demonstrated significant differences in their vascular stiffness, blood pressure and molecular blood profiles compared to women without PE. Consequently, PE has been suggested as a new factor to be involved in maternal health risk assessment [37, 39–41].

Whereas PE & GAH were characteristic for group 3 (overweight), foetal growth restriction and antenatal mortality was typical for group 1 (low BMI). To this end, specifically FGR attracts a lot of attention of clinical research, due to steadily increasing incidence of this complication and still poorly understood mechanisms and evident deficits in targeted prevention [42–44].

Finally, we found significant deviations in maternal and foetal weight gain in both groups 1 (statistically significant insufficient maternal weight gain linked to frequently monitored foetal hypotrophy) and group 3 (statistically significant excessive maternal weight gain linked to frequently monitored foetal hypertrophy) compared to group 2 (standard BMI range). To this end, it has been demonstrated that infants with both excessive and reduced BMI are at increased risk of cardiovascular deficits recorded later on in adulthood; moreover, at particular risk are infants with reduced BMI [45, 46].

Contextually, investigated here maternal health status reflected in all parameters measured, is decisive for both potential maternal/foetal complications in pregnancy and long-term postnatal outcomes. This conclusion is well in consensus with other studies [33, 34, 36, 47] considering maternal vascular status in pregnancy as a powerful indicator and predictor of maternal and foetal health later on in life. Further, newborn weight was positively correlated with the parasympathetic nervous system (PNS) index as well as with nitric oxide levels, heart rate variability and negatively correlated with vascular stiffness.

## Conclusions and expert recommendations in the framework of 3P medicine

During pregnancy, maternal cardiovascular system is exposed to highly increased haemodynamic stress which may overload maternal health in sub-optimal conditions overlooked in pre-pregnancy time. If not protected against consequent damage, progressive abnormalities can be expected in the foetal development and maternal health status during pregnancy and postpartum. Contextually, there are two main concepts to follow in the framework of 3P medicine, namely to develop.

1. advanced screening of sub-optimal health in young populations to predict and prevent individual health risks prior to planned pregnancies
2. in-depth companion diagnostics during pregnancy to predict and prevent long-lasting postpartum health risks of the mother and offspring.

Data collected in the study supports the working hypothesis by demonstrating

- group-specific complications to the health of mother and offspring
- clinical relevance of BMI-related phenotyping in pre-pregnant mothers and
- the fact that maternal cardiovascular maladaptation measured impacts maternal and offspring’s health during pregnancy and may indicate lifelong health risks.

Further, diagnostic approach proposed in the study revealed of great clinical utility synergies between cardiovascular maladaptation and connective tissue dysfunction. To this end, co-diagnosed pre-pregnancy low BMI, pronounced connective tissue dysfunction, increased stiffness of peripheral vessels and decreased blood pressure are considered a highly specific maternal phenotype useful for novel screening programmes in young populations to predict and prevent severe health risks of mother and offspring. This crucial discovery brings together systemic effects characteristic, for example, for individuals with FS phenotype, all potentially linked to connective tissue dysfunction and primary vascular dysregulation with increased risks of myopia, normal tension glaucoma, small vessel disease, ischemic stroke at young age, mitral valve prolapse, impaired wound healing and pathological scarring, cancer and metastatic disease and complications in pregnancy, amongst others [2, 3, 10, 12, 22–26].

In summary, proposed maternal phenotyping is instrumental to predict individual maternal health risks associated with pregnancy and to protect mother and offspring against complications associated with individually detected risk factors. Corresponding screening programmes focused on sub-optimal health in young populations to predict and to mitigate individual health risks prior to pregnancy is an essential innovation for health policy.

### Overlooked subtle symptoms and signs potentially linked to health risks of the mother and offspring during pregnancy and postpartum

1. Hormonal and cardiovascular stress overload caused by pregnancy may provoke health-to-disease transition in individuals with sub-optimal health conditions predisposed to cardiovascular, neurological, neurodegenerative and malignant pathologies. To this end, specialised surveys and comprehensive check-up utilising in
2. Innovative molecular biological tests are strongly recommended [2, 4, 48].
3. There are strong individual deviations reported regarding optimal BMI [3]. Data collected in this study demonstrates that the group with the standard pre-pregnancy BMI-range to certain extent is at risk of complications specific for both low BMI and high BMI groups. This means that the standard pre-pregnancy BMI is not a guarantee for uncomplicated pregnancy course — subtle deviations to health status should be taken into consideration followed by personalised mitigating and protective measures applied.
4. Increased risk of preeclampsia and gestational arterial hypertension is well known for overweight women. Pathomechanisms of PE & GAH in women with normal and low BMI are explored to much less extent. Their pre-pregnancy check-up should consider slightly enhanced vascular stiffness potentially caused by stress-related increase in endothelin-1 blood plasma concentrations [4].
5. Complications linked to phenotypically well-detectable hereditary connective tissue disorders are well acknowledged in the literature. However, minor connective tissue dysfunction (non-specific connective tissue disease, NCTD) with light symptoms is difficult to diagnose. Therefore, NCTD is frequently overlooked as a strong indicator of systemic effects such as endothelial dysfunction, impaired vasodilatation and low-grade inflammation potentially resulting in moderate to severe health risks [49, 50].
6. Increasingly recognised in the scientific community, Flammer syndrome phenotype demonstrates clearly diagnosable symptoms and behavioural patterns of great clinical utility. Phenotype-specific features such as low BMI, disturbed microcirculation, increased endothelin-1 level, stress overload, compromised mitochondrial health and potential connective tissue dysfunction, amongst others — all are relevant for systemic effects, sub-optimal health conditions and potential complications in pregnancy and postpartum [10, 12]. Noteworthy, current study demonstrated no cases of PE/GAH/GD in the cohort of pregnant women with FS phenotype. Further, current study confirmed that FS phenotype may be strongly predisposed to oligohydramnios (see the “In-depth analysis of individual cases” section) as discovered earlier [12]. Compared with women with normal values of the amniotic fluid index, otherwise healthy pregnant women diagnosed with oligohydramnios demonstrate significantly higher rates of an infant with meconium aspiration syndrome, cesarean delivery for fetal distress and admission to the neonatal intensive care unit [51]. Oligohydramnios may be further associated with the placental-driven foetal growth restriction, due to deficient remodelling of the uterine spiral arteries supplying the placenta during early pregnancy. Resulting mal-perfusion may stress placental tissues, suppress protein synthesis and reduce cell proliferation. This reduces the surface area for maternal–fetal exchange and dysregulate gene expression affecting placental transport, and endocrine, metabolic and immune functions [52]. High level of stress may activate proinflammatory and apoptotic pathways contributing to maternal endothelial dysfunction and ischemic-hypoxic lesions characteristic for individuals with FS phenotype [12]. Flammer syndrome phenotype is well diagnosable in puberty [11]. Consequently, personalised health risk assessment in individuals with FS phenotype is feasible to be performed early in life.

**Fig. 3:**
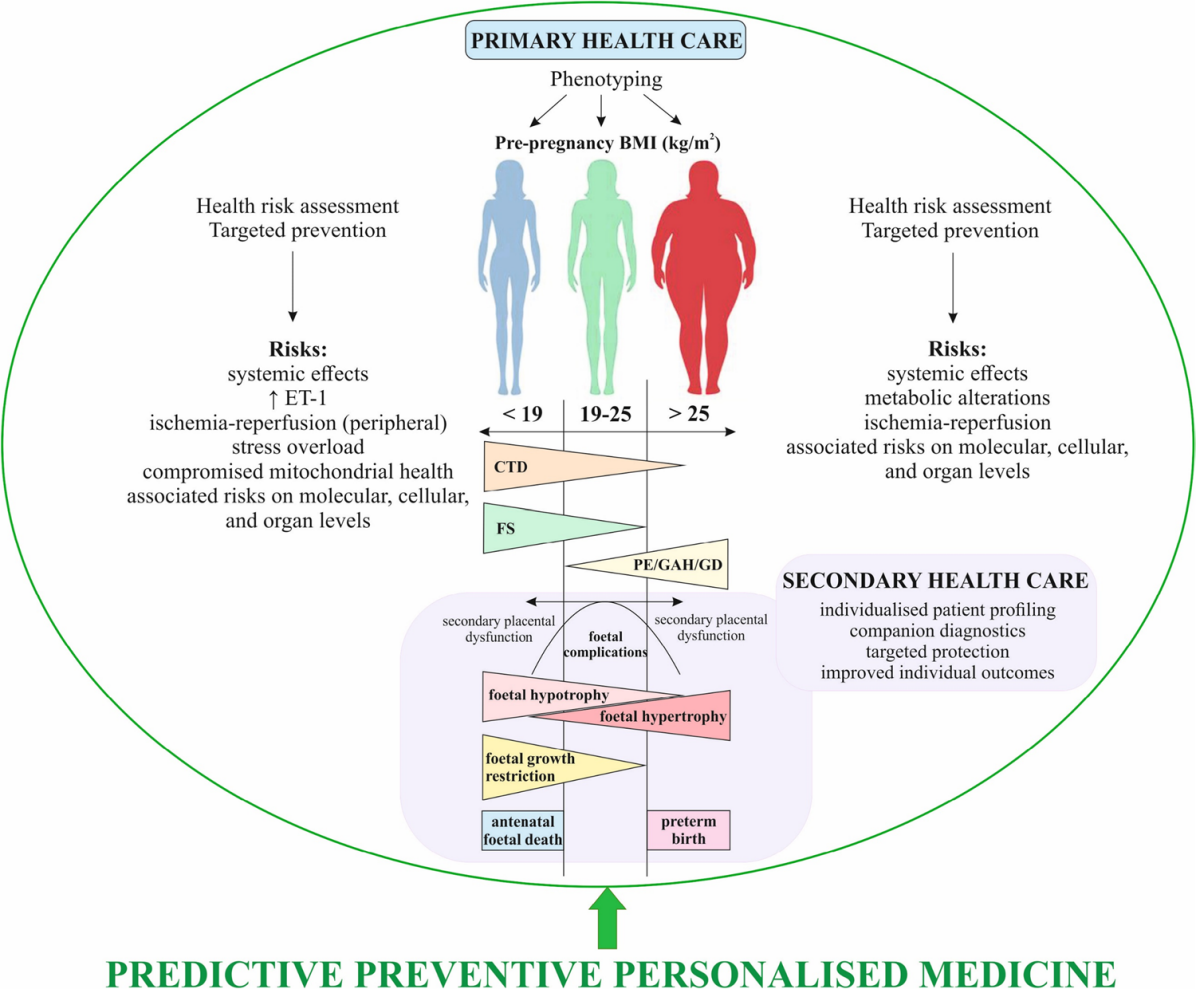
Pregnancy-associated health risks to mother and offspring identified in groups of comparison; PPPM approach based on pre-pregnancy phenotyping of the mother, health risk assessment and individualised protective and mitigation measures proposed for primary and secondary care; Abbreviations: < –increasing prevalence across the groups 1–3; > –decreasing prevalence across the groups 1–3; FS, Flammer syndrome; CTD, connective tissues dysfunction; PE, preeclampsia; GAH, gestational arterial hypertension; GD, gestational diabetes; ET-1, enddothelin-1

Main outcomes are summarised in Fig. 3.

### Recommendations for individualised mitigation measures and protection to health of the affected mother and offspring — application of natural substances in primary and secondary care

Individualised dietary patterns composed before, during and after pregnancy may be health protective for both the mother and offspring [53, 54]. Based on the available evidence, herewith we exemplify protective effects of natural substances adapted to individual risks listed below.

#### Oxidative stress

There are evident beneficiary effects attributed to the *Mediterranean diet* (MD) characterised by high intake of fruits, vegetables, whole grain cereals, fish, legumes and nuts accompanied with a limited consumption of red meat [55]. Recent studies demonstrate that MD leads to a significant stress reduction resulting in reduced risks of hypotrophy in newborns [56]. Antenatal supplementation with antioxidant vitamins (including beta-carotene, vitamins) reduces oxidative stress at delivery in mothers at risk of preterm delivery and neonates [57]. Further, pomegranate juice which is rich in polyphenols demonstrates a potential to reduce placental oxidative stress [58].

#### Endothelial dysfunction

Endothelial dysfunction can be caused by increased blood levels of the vasoconstrictor endothelin-1 (ET-1) with far-reaching consequences [4]. Elevated ET-1 levels are characteristic for individuals with Flammer syndrome phenotype. Phytochemicals (e.g. in virgin olive oil, red clover and hop extracts) improving the nitric oxide release demonstrate potential to increase vasodilatation mitigating thereby the endothelial dysfunction related to high ET-1 [59, 60]. Further, the anti-inflammatory and cardioprotective properties of curcumin (a natural polyphenolic flavonoid isolated from the rhizomes of Curcuma longa) are evidently linked to the Ca^2+^/NFATc1-regulated release of ET-1. This pathway is suppressed by dietary curcumin supplements with a protection potential against cardiovascular risks [61].

Naturally occurring phytochemicals and antioxidants beneficially interfere into pathways involved in the interplay between endothelial dysfunction, increased oxidative stress and preeclampsia risks [62]. Dietary patterns rich in vegetable combined with supervised physical activity patterns decrease risks of pregnancy-induced hypertension [63]. Further, epigallocatechin gallate significantly improves treatment efficacy of oral nifedipine in pregnancy-induced severe preeclampsia [64].

#### Connective tissue dysfunction

There is a reciprocal interrelationship between CTD and pregnancy/outcomes [65]. Contextually, CTD patients are at high risk to be monitored and mitigated in pre-pregnancy time, during pregnancy and in postpartum care [66]. To this end, macronutrients and micronutrients play a multifactorial role in CTD development and severity grad. Several plant extracts and herbal formulas (such as *Camellia sinensis* L. commonly known as green tea, pycnogenol — an extract of *Pinus pinaster Aiton*, or Sophorae Radix) demonstrate evident anti-CTD protection e.g. in case of cutaneous lupus erythematosus [67].

#### Compromised mitochondrial health

Mitochondria play essential role in multiple processes beyond energy production, health status, reproductive health, physiological changes during pregnancy and embryogenesis. Contextually, healthy pregnancy and postpartum requires optimal mitochondrial health and its quality control [68]. Per evidence, nutrients are directly involved in modulation of mitochondrial function that makes mitochondria to the target for dietary regulation [69]. To this end, flavonoids are highly effective in lowering oxidative stress associated with risks for both pregnancy and mitochondrial health [70, 71].

#### Pro-inflammatory conditions

Maternal inflammation could result in metabolic reprogramming at the stage of periconceptional period as well as at other stages of placental/embryonic development [72]. Naturally occurring phytochemicals exert potent anti-inflammatory effects [73]. To this end, consumption of garlic demonstrates a significant decrease in CRP levels as measured, for example in pregnant women at high risk for preeclampsia [74]. Further, silibinin dietary supplement attenuates oxidative metabolism and significantly reduces pro-inflammatory cytokine production by monocytes in pregnant women diagnosed with preeclampsia [75–77].

#### Gestational diabetes

Plant-based diet demonstrates potent protective effects against gestational diabetes beneficial for groups at high risk [78].

#### The timing

The crucial factor for an effective protection against pregnancy-associated risks is the timing. Optimal dietary and lifestyle patterns should be adapted to the individual phenotype in pre-pregnancy period of time. Exemplified with the study presented by Vinter et al., lifestyle intervention in obese women in early pregnancy resulted in no improvements in obstetric and metabolic outcomes [79]. The authors emphasise the importance of the individualised supervision in pre-pregnancy which is crucial for improved pregnancy outcomes and post-partum.

## Data Availability

Not applicable.

## Abbreviations

AIXa: Aortic augmentation index
AIXb: Brachial augmentation index
RWV: Aortic reverse pulse wave velocity
BMI: Body mass index
Ca^2+^/NFATc1: Ca^2+^ / nuclear factor of activated T- ells
CDBP: Central diastolic blood pressure
CRP: C-reactive protein
CSBP: Central systolic blood pressure
CTD: Connective tissue dysfunction
CVD: Cardiovascular disease
ET-1: Enddothelin-1
GAH: Gestational arterial hypertension
GD: Gestational diabetes
FS: Flammer syndrome
MD: Mediterranean diet
mm Hg: Millimetre of mercury
NCTD: Non-specific connective tissue disease
PE: Preeclampsia
PDBP: Peripheral diastolic blood pressure
PNS: Parasympathetic nervous system
PPPM / 3PM: Predictive Preventive Personalised Medicine
PSBP: Peripheral systolic blood pressure
RWVa: Aortic reverse pulse wave velocity
SPs: Study participants
VD: Vascular stiffness

## References

[1] Roth GA, Mensah GA, Johnson CO, Addolorato G, Ammirati E, Baddour LM (2020). Global Burden of cardiovascular diseases and risk factors, 1990–2019: update from the GBD 2019 Study. J Am Coll Cardiol.

[2] Polivka J, Polivka J, Pesta M, Rohan V, Celedova L, Mahajani S (2019). Risks associated with the stroke predisposition at young age: facts and hypotheses in light of individualized predictive and preventive approach. EPMA J.

[3] Golubnitschaja O, Liskova A, Koklesova L, Samec M, Biringer K, Büsselberg D, et al. Caution, “normal” BMI: health risks associated with potentially masked individual underweight-EPMA Position Paper 2021. EPMA J. 2021;1–22. 10.1007/s13167-021-00251-4 10.1007/s13167-021-00251-4 PMC8368050 34422142

[4] Torres Crigna A, Link B, Samec M, Giordano FA, Kubatka P, Golubnitschaja O. Endothelin-1 axes in the framework of predictive, preventive and personalised (3P) medicine. EPMA J. 2021;1–41. 10.1007/s13167-021-00248-z 10.1007/s13167-021-00248-z PMC8334338 34367381

[5] Palinski W (2014). Effect of maternal cardiovascular conditions and risk factors on offspring cardiovascular disease. Circulation.

[6] Creanga AA, Syverson C, Seed K, Callaghan WM (2017). Pregnancy-Related mortality in the United States, 2011–2013. Obstet Gynecol.

[7] Perak AM, Lancki N, Kuang A, Labarthe DR, Allen NB, Shah SH (2021). Associations of maternal cardiovascular health in pregnancy with offspring cardiovascular health in early adolescence. JAMA.

[8] Mszar R, Gopal DJ, Chowdary R, Smith CL, Dolin CD, Irwin ML (2020). Racial/ethnic disparities in screening for and awareness of high cholesterol among pregnant women receiving prenatal care. J Am Heart Assoc.

[9] Pétursdóttir Maack H, Larsson A, Axelsson O, Olovsson M, Wikström A-K, Sundström PI (2020). Pregnancy in metabolic healthy and unhealthy obese women. Acta Obstet Gynecol Scand.

[10] Golubnitschaja O, editor. Flammer syndrome: from phenotype to associated pathologies, prediction, prevention and personalisation; Advances in Predictive, Preventive and Personalised Medicine; Springer International Publishing: Cham, 2019; Vol. 11; ISBN 978–3–030–13549–2.

[11] Konieczka K, Ritch R, Traverso CE, Kim DM, Kook MS, Gallino A (2014). Flammer syndrome. EPMA J.

[12] Golubnitschaja O, Flammer J (2018). Individualised patient profile: clinical utility of Flammer syndrome phenotype and general lessons for predictive, preventive and personalised medicine. EPMA J.

[13] Polivka J, Altun I, Golubnitschaja O (2018). Pregnancy-associated breast cancer: the risky status quo and new concepts of predictive medicine. EPMA J.

[14] Merlocco A, Lacro RV, Gauvreau K, Rabideau N, Singh MN, Prakash A (2017). Longitudinal Changes in segmental aortic stiffness determined by cardiac magnetic resonance in children and young adults with connective tissue disorders (the Marfan, Loeys-Dietz, and Ehlers-Danlos syndromes, and nonspecific connective Tissue Disorders). Am J Cardiol.

[15] Klemenov AV (2015). Hereditary connective tissue disorders: nomenclature and diagnostic algorithm. Klinicist.

[16] Omboni S, Posokhov IN, Parati G, Avolio A, Rogoza AN, Kotovskaya YV (2016). Vascular health assessment of the hypertensive patients (VASOTENS) Registry: Study Protocol of an International, Web-Based Telemonitoring Registry for Ambulatory Blood Pressure and Arterial Stiffness. JMIR Res Protoc.

[17] Bello NA, Woolley JJ, Cleary KL, Falzon L, Alpert BS, Oparil S (2018). Accuracy of blood pressure measurement devices in pregnancy. Hypertension. Am Heart Assoc.

[18] Bartosh LF, Dorogova JV, Kuznecova TN, Krylova AV (2006). The testing of BPLab ambulatory blood pressure monitor on the pregnant in conformity with International Protocol of the European Society of Hypertension (ESH-2001). Arter Gipertenz.

[19] Penpattharakul W, Pithukpakorn M (2016). Revised Ghent Criteria is comparable to original diagnostic criteria for marfan syndrome with increased ability to clinically diagnose related disorders. J Med Assoc Thai.

[20] Duggal P, Petri WA (2018). Does malnutrition have a genetic component?. Annu Rev Genomics Hum Genet.

[21] Prakash A, Adlakha H, Rabideau N, Hass CJ, Morris SA, Geva T (2015). Segmental aortic stiffness in children and young adults with connective tissue disorders: relationships with age, aortic size, rate of dilation, and surgical root replacement. Circulation.

[22] Bubnov R, Polivka J, Zubor P, Konieczka K, Golubnitschaja O (2017). “Pre-metastatic niches” in breast cancer: are they created by or prior to the tumour onset? “Flammer Syndrome” relevance to address the question. EPMA J.

[23] Gunawardane ND, Dontsi M, Lyon LL (2020). Risk of non-melanoma skin cancer in connective tissue disease and the impact of immunosuppressive therapy. J Drugs Dermatol.

[24] Shao W, Zhou Q, Tang X (2020). Current and emerging treatment options for lung cancer in patients with pre-existing connective tissue disease. Pulm Pharmacol Ther.

[25] Chu C-Y, Chang C-C, Prakash E, Kuo M-L (2008). Connective tissue growth factor (CTGF) and cancer progression. J Biomed Sci.

[26] Karppinen S-M, Heljasvaara R, Gullberg D, Tasanen K, Pihlajaniemi T. Toward understanding scarless skin wound healing and pathological scarring. F1000Res. 2019;8:F1000 Faculty Rev–787. 10.12688/f1000research.18293.1 10.12688/f1000research.18293.1 PMC6556993 31231509

[27] Aroor AR, Jia G, Sowers JR (2018). Cellular mechanisms underlying obesity-induced arterial stiffness. Am J Physiol Regul Integr Comp Physiol.

[28] Fuster JJ, Ouchi N, Gokce N, Walsh K (2016). Obesity-Induced changes in adipose tissue microenvironment and their impact on cardiovascular disease. Circ Res.

[29] Para I, Albu A, Porojan MD (2021). Adipokines and arterial stiffness in obesity. Medicina (Kaunas).

[30] Sabbatini AR, Fontana V, Laurent S, Moreno H (2015). An update on the role of adipokines in arterial stiffness and hypertension. J Hypertens..

[31] Nakamura K, Fuster JJ, Walsh K (2014). Adipokines: a link between obesity and cardiovascular disease. J Cardiol.

[32] Nuckols VR, Holwerda SW, Luehrs RE, DuBose LE, Stroud AK, Brandt D (2020). Beat-to-beat blood pressure variability in the first trimester is associated with the development of preeclampsia in a prospective cohort: relation with aortic stiffness. Hypertension.

[33] Rueangjaroen P, Luewan S, Phrommintikul A, Leemasawat K, Tongsong T (2021). The cardio-ankle vascular index as a predictor of adverse pregnancy outcomes. J Hypertens.

[34] Turi V, Iurciuc S, Crețu OM, Tit DM, Bungau S, Apostol A (2021). Arterial function in hypertensive pregnant women. Is arterial stiffness a marker for the outcomes in pregnancy?. Life Sci.

[35] Phan K, Schiller I, Dendukuri N, Gomez Y-H, Gorgui J, El-Messidi A (2021). A longitudinal analysis of arterial stiffness and wave reflection in preeclampsia: Identification of changepoints. Metabolism.

[36] Pereira MM, Torrado J, Sosa C, Zócalo Y, Bia D (2021). Role of arterial impairment in preeclampsia: should the paradigm shift?. Am J Physiol Heart Circ Physiol.

[37] Kim S, Lim HJ, Kim J-R, Oh KJ, Hong J-S, Suh J-W (2020). Longitudinal change in arterial stiffness after delivery in women with preeclampsia and normotension: a prospective cohort study. BMC Pregnancy Childbirth.

[38] Karatza AA, Dimitriou G (2020). Preeclampsia Emerging as a novel risk factor for cardiovascular disease in the offspring. Curr Pediatr Rev.

[39] Witvrouwen I, Mannaerts D, Ratajczak J, Boeren E, Faes E, Van Craenenbroeck AH (2021). MicroRNAs targeting VEGF are related to vascular dysfunction in preeclampsia. Biosci Rep..

[40] Powe CE, Levine RJ, Karumanchi SA (2011). Preeclampsia, a disease of the maternal endothelium: the role of antiangiogenic factors and implications for later cardiovascular disease. Circulation.

[41] Lisowska M, Pietrucha T, Sakowicz A (2018). Preeclampsia and related cardiovascular risk: common genetic background. Curr Hypertens Rep.

[42] Anca A, Horhoianu V, Horhoianu I (2014). Predictive factors for intrauterine growth restriction. J Med Life.

[43] ACOG (2013). Practice bulletin no. 134: fetal growth restriction. Obstet Gynecol.

[44] Dall’Asta A, Brunelli V, Prefumo F, Frusca T, Lees CC (2017). Early onset fetal growth restriction. Matern Health Neonatol Perinatol.

[45] Hughes MM, Black RE, Katz J (2017). 2500-g low birth weight cutoff: history and implications for future research and policy. Matern Child Health J..

[46] Nakano Y (2020). Adult-onset diseases in low birth weight infants association with adipose tissue maldevelopment. J Atheroscler Thromb.

[47] Benvenuto S, JooTuroni C, Marañón RO, Chahla R, Peral de Bruno M (2022). Changes in vascular function and autonomic balance during the first trimester of pregnancy and its relationship with the new-born weight. J Obstet Gynaecol.

[48] Crigna AT, Samec M, Koklesova L, Liskova A, Giordano FA, Kubatka P, et al. Cell-free nucleic acid patterns in disease prediction and monitoring-hype or hope? EPMA J. 2020; 1–25 10.1007/s13167-020-00226-x 10.1007/s13167-020-00226-x PMC7594983 33144898

[49] Ateka-Barrutia O, Nelson-Piercy C (2013). Connective tissue disease in pregnancy. Clin Med (Lond)..

[50] Bitar C, Chan MP (2021). Connective tissue diseases in the skin: emerging concepts and updates on molecular and immune drivers of disease. Surg Pathol Clin..

[51] Rabie N, Magann E, Steelman S, Ounpraseuth S (2017). Oligohydramnios in complicated and uncomplicated pregnancy: a systematic review and meta-analysis. Ultrasound Obstet Gynecol..

[52] Burton GJ, Jauniaux E (2018). Pathophysiology of placental-derived fetal growth restriction. Am J Obstet Gynecol..

[53] Lim SX, Loy SL, Colega MT, Lai JS, Godfrey KM, Lee YS (2022). Prepregnancy adherence to plant-based diet indices and exploratory dietary patterns in relation to fecundability. Am J Clin Nutr..

[54] Pistollato F, Sumalla Cano S, Elio I, Masias Vergara M, Giampieri F, Battino M (2015). Plant-based and plant-rich diet patterns during gestation: beneficial effects and possible shortcomings12. Adv Nutr.

[55] Amati F, Hassounah S, Swaka A (2019). The impact of mediterranean dietary patterns during pregnancy on maternal and offspring health. Nutrients.

[56] Crovetto F, Crispi F, Casas R, Martín-Asuero A, Borràs R, Vieta E (2021). Effects of mediterranean diet or mindfulness-based stress reduction on prevention of small-for-gestational age birth weights in newborns born to at-risk pregnant individuals: The IMPACT BCN Randomized Clinical Trial. JAMA.

[57] Bolisetty S, Naidoo D, Lui K, Koh THHG, Watson D, Whitehall J (2002). Antenatal supplementation of antioxidant vitamins to reduce the oxidative stress at delivery--a pilot study. Early Hum Dev.

[58] Chen B, Tuuli MG, Longtine MS, Shin JS, Lawrence R, Inder T (2012). Pomegranate juice and punicalagin attenuate oxidative stress and apoptosis in human placenta and in human placental trophoblasts. Am J Physiol Endocrinol Metab..

[59] Sanchez-Rodriguez E, Lima-Cabello E, Biel-Glesson S, Fernandez-Navarro JR, Calleja MA, Roca M (2018). Effects of virgin olive oils differing in their bioactive compound contents on metabolic syndrome and endothelial functional risk biomarkers in healthy adults: a randomized double-blind controlled trial. Nutrients.

[60] Jeon SY, Kim MR, Lee EO, Jeon BH, Lee JJ, Lee YC (2020). Effect of a new herbal composition comprised of red clover and hop extract on human endothelial cell damage and vasorelaxant activity. J Food Biochem.

[61] Hernández M, Wicz S, Santamaría MH, Corral RS (2018). Curcumin exerts anti-inflammatory and vasoprotective effects through amelioration of NFAT-dependent endothelin-1 production in mice with acute Chagas cardiomyopathy. Mem Inst Oswaldo Cruz..

[62] Sánchez-Aranguren LC, Prada CE, Riaño-Medina CE, Lopez M (2014). Endothelial dysfunction and preeclampsia: role of oxidative stress. Front Physiol.

[63] Longo-Mbenza B, Kadima-Tshimanga B, Buassa-bu-Tsumbu B, M’buyamba K (2008). Diets rich in vegetables and physical activity are associated with a decreased risk of pregnancy induced hypertension among rural women from Kimpese. DR Congo. Niger J Med.

[64] Shi D-D, Guo J-J, Zhou L, Wang N (2018). Epigallocatechin gallate enhances treatment efficacy of oral nifedipine against pregnancy-induced severe pre-eclampsia: a double-blind, randomized and placebo-controlled clinical study. J Clin Pharm Ther.

[65] Ryan SL, Bhattacharyya S (2019). Connective tissue disorders in pregnancy. Neurol Clin.

[66] Saar P, Hermann W, Müller-Ladner U (2006). Connective tissue diseases and pregnancy. Rheumatol (Oxford).

[67] Lubov JE, Jamison AS, Baltich Nelson B, Amudzi AA, Haas KN, Richmond JM (2022). Medicinal plant extracts and natural compounds for the treatment of cutaneous lupus erythematosus: a systematic review. Front Pharmacol.

[68] Koklesova L, Mazurakova A, Samec M, Kudela E, Biringer K, Kubatka P (2022). Mitochondrial health quality control: measurements and interpretation in the framework of predictive, preventive, and personalized medicine. EPMA J..

[69] Rodríguez-Cano AM, Calzada-Mendoza CC, Estrada-Gutierrez G, Mendoza-Ortega JA, Perichart-Perera O (2020). Nutrients, mitochondrial function, and perinatal health. Nutrients..

[70] Ebegboni VJ, Dickenson JM, Sivasubramaniam SD (2019). Antioxidative effects of flavonoids and their metabolites against hypoxia/reoxygenation-induced oxidative stress in a human first trimester trophoblast cell line. Food Chem.

[71] Cao Y, Zhao H, Wang Z, Zhang C, Bian Y, Liu X (2020). Quercetin promotes in vitro maturation of oocytes from humans and aged mice. Cell Death Dis Nature Publ Group.

[72] Parisi F, Milazzo R, Savasi VM, Cetin I (2021). Maternal low-grade chronic inflammation and intrauterine programming of health and disease. Int J Mol Sci Multidiscip Digit Publ Inst.

[73] Kubatka P, Mazurakova A, Samec M, Koklesova L, Zhai K, AL-Ishaq R (2021). Flavonoids against non-physiologic inflammation attributed to cancer initiation, development, and progression—3PM pathways. EPMA J.

[74] Aalami-Harandi R, Karamali M, Asemi Z (2015). The favorable effects of garlic intake on metabolic profiles, hs-CRP, biomarkers of oxidative stress and pregnancy outcomes in pregnant women at risk for pre-eclampsia randomized, double-blind, placebo-controlled trial. J Matern Fetal Neonatal Med.

[75] Matias ML, Gomes VJ, Romao-Veiga M, Ribeiro VR, Nunes PR, Romagnoli GG (2019). Silibinin downregulates the NF-κB pathway and NLRP1/NLRP3 Inflammasomes in monocytes from pregnant women with preeclampsia. Molecules.

[76] Cristofalo R, Bannwart-Castro CF, Magalhães CG, Borges VTM, Peraçoli JC, Witkin SS (2013). Silibinin attenuates oxidative metabolism and cytokine production by monocytes from preeclamptic women. Free Radic Res.

[77] Giorgi VSI, Peracoli MTS, Peracoli JC, Witkin SS, Bannwart-Castro CF (2012). Silibinin modulates the NF-κb pathway and pro-inflammatory cytokine production by mononuclear cells from preeclamptic women. J Reprod Immunol.

[78] Chen Z, Qian F, Liu G, Li M, Voortman T, Tobias DK (2021). Prepregnancy plant-based diets and the risk of gestational diabetes mellitus: a prospective cohort study of 14,926 women. Am J Clin Nutr.

[79] Vinter CA, Tanvig MH, Christensen MH, Ovesen PG, Jørgensen JS, Andersen MS (2018). Lifestyle intervention in Danish Obese pregnant women with early gestational diabetes mellitus according to WHO 2013 Criteria does not change pregnancy outcomes: results from the LiP (Lifestyle in Pregnancy) Study. Diabetes Care.

